# Medical Items and News of Interest to the Physician

**Published:** 1880-04

**Authors:** 


### For the Use of Physicians.

CULLED FROM THE STANDARD MEDICAL JOURNALS OF THE WORLD.

NOTE.—These formulæ are intended only for the regular practitioner of medicine, and should be employed only by him, or under his immediate supervision.

**Mr. Soelberg Welles.**

The great English oculist, and author of "The Diseases of the Eye and their Treatment," died at Cannes, recently.

**Nausea.**

Dr. Bussey, (*American Practitioner*) recommends the combination of oxalate of cerium with opium to obviate nausea,, etc. He also gives strong coffee or the citrate of caffeine.

**Quinia and Iron Tonic.**

℞

| | |
|---|---|
| Ferri et quiniæ citr, | grains XL. |
| Acidi hydrobrom, | fl. ʒ ij. |
| Spirit limon, | fl. ʒ j. |
| Syrupi, | fl. ʒ vj. |
| Aquæ, | q. s. ad. fl. ℥ vj. |

Dose: ½ to 1 fl. ℥.—*Pharm. Journ.*

**Cracked Nipples.**

Dr. Haussmann treats cracked nipples by applying lint soaked in a two-per-cent. solution of carbolic acid. The wet lint should be applied every two or three hours. The treatment gives instant relief from pain, and although the child continues to use the nipples, cure is established within three days.

**Tasteless Saline Purgatives.**

In the *Paris Medical*, Dr. Yoon recommends the following combination as almost concealing the disagreeable taste of Epsom salts :

℞

Magnesii sulphatis, ʒ v.

Essentiæ menthæ, gtt. iij.

M. To be dispensed in a very little water.

**Prostatic Enlargement.**

Ergot internally is strongly advocated in this complaint by Mr. R. Harrison, in the *Lancet*. He sums up all treatment by saying that the great point to aim at is to secure healthy urine, and this is attained by tying in an elastic catheter, and allowing the urine to pass away as it is secreted, until it becomes healthy, and then draw it off at regular and gradually prolonged intervals.

**Diabetes.**

Dr. Schætzke publishes in the Berliner klin. Wochenschr, a translation which we find in the *British Med. Jour.*, three cases of diabetes successfully treated with salicylic acid. It was administered in 3 gramme doses (45½ grains) three times a day.

**Ergot in Catarrhal Affections.**

Ergot is now recommended as a local remedy in catarrhal affections of the eye and throat. In chronic conjunctivitis the strength is 65 of the extract to 32 of water, a little glycerin being added to preserve the drug. In throat affections, it forms an excellent element in a gargle, or may be applied in combination with tincture of iodine. In nasal catarrh, it may be applied by means of gelatin bougies.

**Neuralgia.**

In neuralgia of the fifth pair, Prof. Fereol, of Lyons, believes he can report good results from the following :

℞

Cupri ammonio-sulphatis, gr. ij–iij.

Syrupi florum aurant, f. ʒ j.

Aquæ distill., f. ℥ iij.

One-third at a dose, three times a day.

It gives a fetid odor to the breath, which should be disregarded.

**Ovarian Dismenorrhœa.**

The following combinination is well spoken of in the *Medical Brief*, by Dr. Pattee:

℞

Tinct. Pulsatillæ,

Tinct. acteæ albæ,

Tinct. cimicifugæ, āā gtt. xv.

Aquæ, f. ℥ iv.

A teaspoonful three or four times a day.

**Nausea of Pregnancy.**

Dr. F. A. Burrall, of this city, writes : "One of the best remedies for the nausea which attends the parturient state is the quickly roasted grain of the *Zea mays* or Indian corn. It is too familiarly known as 'pop-corn' to require any discription. Many physicians are not aware of the beneficial results which may be derived from the use of this simple agent. It should be white and light, and may be eaten freely, sprinkled with salt. I think it is no exaggeration to say that it will be found of the greatese service in many cases where the products of the chemist's art have proved unavailing."—*The Medical Record.*

**Painful Sores.**

Archambault recommends the following mixture as a soothing application to painful sores of any kind, particularly in children:

℞

Mucilag. Cydoniæ, 15 gm.

Extracti Krameriæ, 25 gm.

Mix.

**Precaution in Administering Iron.**

Dr. T. Grainger Stewart, has discovered that when, during the administration of the tincture of chloride of iron, functional derangements of the stomach and liver arises with furred tongue, impaired appetite, headache, etc., these symptoms rapidly disappear upon adding one-half grain of the chloride of ammonium to each minim of the tincture. He finds this combination notably useful in cases of heart disease accompanied by anæmia and debility.

**Sea-Sickness.**

Dr. Geo. M. Beard, during a recent trip to Europe, experimented somewhat in the treatment of sea-sickness, and found that large doses of the bromides—of sodium perferably—with caffein, and hypodermic doses of sulphate of atropia, were effectual means of controlling the trouble. Bromides to the extent of 30, 60, or 90 grains daily, begun two or three days before sailing and continued while at sea, deprived sea-sickness of one-half of its terrors, and in that respect verified the claims made by Dr. F. D. Lente, for the same remedy.—*Medical Record.*

**Cystitis in the Male.**

The following is frequently employed at Jefferson Medical College Hospital:

℞

Pulv. opii, grs. xij.

Camphoræ, grs. xxx.

Ext. belladonnæ, grs. iij.

Ol. theobromæ, q. s.

M. et div. in suppos. no. vi. Sig.: One each night before retiring.

Dr. S. W. Gross' prescription is also often employed. It is as follows:

℞

Copaibæ, ʒ iv.

Acidi benzoici, ℈ iv.

Acaciæ,

Sacchari, aa ʒ ij.

Olei gaultheriæ, gtt. xx.

Aq. camph., q. s. ad., ℥ viij.

M. Sig.; A tablespoonful every four hours. —*Medical Record.*

**Antiscorbutic Tincture.**

| | |
|---|---|
| Tincture of catechu, | 25 parts. |
| " myrrh, | 15 " |
| " cinchona, | 10 " |
| Balsam Peru, | 1½ part. |
| Spirit of cochlearia, | 10 parts. |
| Diluted alcohol, | 20 " |

M. A teaspoonful in warm water; rinse the mouth and rub the gums.

**To Make Leeches Bite.**

Dr. Alex. Hadden, of this city, writes us that the secretions of the skin of some persons are obnoxious to leeches, and prevent their biting when they are applied. This may be remedied by first washing the surface with a weak solution of bicarbonate of soda and then drying it thoroughly with a clean towel, after which failure will be rare.—*Pharm. Jour.*

**Whooping Cough.**

In pertussis, Dr. Pollak, of Austria, recommends, for insufflation:

℞

Quiniæ tannatis,
Sodii bicarbonatis, āā 5 parts.
Pulv. acaciæ, 100 parts.

Use with an insufflator.—*Med. & Surg. Reporter.*

**Eczema Intertrigo of Infants.**

℞

Plumbi acetatis, gr. xxx.
Acidi acetici diluti, ʒ ij.
Glycerinæ, ℥ iss.
Aquæ rosæ, ad ℥ viij.

M. Wash the sore parts well with soap and water, dry carefully, then apply the above.

Dr. H. B. Hodges writes the *British Medical Journal*, that in hundreds of cases, during a quarter of a century of practice, he never knew the above to fail to cure the disease. He uses no internal medication.

**Prevention of Mammary Abcess**

Dr. Jamieson says that distension of the milk-ducts from inflammation due to cold caught in early lactation, imperfect formation of the nipple, fissured nipple or localized hyperemias, from constant suckling in anæmic and feeble persons, are the usual causes of mammary abscess. He urges the use of low-cut corsets; wide, easy dresses; attention to drawing out and developing the nipples from the beginning of conception, in addition to the usual means in use after delivery.—*Louisville Medical News.*

**Simple Glaucoma.**

M. de Wecker advocated before the French Ass'n of Science, recently, a new operation for arresting simple glaucoma. The operation which he advocates is sclerotomy, entering in to extensive details as to the manner of performing it. He has performed 48 operations and congratulates himself upon the results obtained. He recommends it as a substitute for iridectomy.

---

**Scabies.**

Dr. Liveing, in the *British Medical Journal*, believes it to be a common error in applying sulphur ointment too strong and continuing it too long. Nothing is more effectual than the sulphur ointment, of the pharmacopœia if applied half its strength. He recommends that it be thoroughly rubbed in to the parts affected every night for three nights. A rest then for a week, and an application for two or three nights longer, will invariably cure.

---

**Dog Fennel.**

One of the camomile species, growing in the back lanes of our towns throughout the South, possesses the stomachic and tonic properties of the camomile of the shops. In addition the leaves of the plant contain a principle almost identical with cantharidine. When bruised and applied to the skin they produce prompt and active epispastic effect.—*Southern Med. Record.*

---

**Habitual Constipation.**

Dr. G. W. Cook, of Syracuse, N. Y., recommends, in an article in *New Preparations*, a new medicine for this troublesome ailment. He has had excellent success in the administration of *cascara sagrada*, administered as follows:

℞

Fld. ext. cascara sagrada,
Simple syrup, āā, f. ℥ j;
Ext. malt, f. ℥ ij. M.

Sig: A teaspoonful before meals.

---

**Visceral Disorders.**

Vidal has recently laid down the rule that in visceral disorders, the special tender points are usually as follows: In ulcer of the stomach the tender point is over the spinous process of the sixth dorsal vertebra, and in liver disorder it is over the fourth. In several cases of perityphlitis he found a tender point at the junction of the second and third dorsal verebræ.—*Chicago Med. Gazette.*

**Acute Coryza.**

R. Rudolph reports enclyptus as a specific for acute coryza, and declares that chewing the twigs and swallowing saliva secreted will result in rapid relief. His experience includes experiments upon his patients and upon himself. It is probable that the fluid extract would be equally efficacious.

---

**Urticaria.**

Bromide of Potassium in solution, one ounce to a pint of water, used externally, will almost invariably allay the irritation of urticaria simplex. This has been the experience of Dr. E. C. Dudley, of Chicago, who has prescribed it frequently and seldom been disappointed in its results. It appears to act as a local sedative to the vaso-motor nerves of the skin.

---

**Fissured Nipples.**

Dr. E. O'Hara, of the Obstetrical Society of Philadelphia, has found the following application to fissured nipples very satisfactory, and giving prompt relief to pain:

℞

Iodoform, ʒ ss.

Collodion, ℥ j.

M. Sig.—Paint on the part from time to time.

---

**Fluid Extract Grindelia Squarrosa.**

I was called, August 26, to see Mr. G. S., aged forty-three, a coal miner. He suffered for over one year with constantly recurring intermittent fever, sometimes broken with quinine and Fowler's solution, but returning while taking it, I ordered him one and one-half ounces of grindelia squarrosa, to be taken thirty drops three times a day after meals, in water. He has not chilled since. I might record a number of cases in which this remedy has been used with success as a substitute for quinine. I would be pleased if the doctors would give it a trial and report through the *Brief.—L. C. McDowell M. D., in Medical Brief.*

---

**Obesity.**

A medical man in an English town lost eight pounds in three weeks, one and a half pounds in the next three weeks, and after twelve weeks found himself thirteen pounds lighter. It is only fair to say that he strictly dieted himself, avoiding butter, sugar, beer, etc.; but this treatment he had tried by itself before without noticing much difference in his weight. In another case a gentleman lost eight pounds in six weeks without any change of his diet. A lady lost more than twenty pounds in nine weeks, also without any particular change of diet. The above all took the fluid extract of fuous vesiculosus, and found no ill effects on the general health. I can hear no complaints of diarrhœa, excessive micturition, nor smelling or sweating of the feet.—*Dr. Fairbank, in British Medical Journal.*

---

**Diphtheria.**

Mr. Henry Bergeron has obtained very favorable results from inhalations of an atmosphere containing a definite small proportion of hydrofluoric acid gas. The quantity employed is 1 gram for each cubic metre (1.3 cubic yards) of air space in the room during 3 hours. The gas is generated by allowing the proper amount of acid slowly to evaporate from a vessel (of lead) placed on a table a short distance from the bed of the patient.—*Le Monit. Therap.*,

---

**Trichinæ.**

Dr. Hæberlein reports that an apothecary in Crailsheim, Mr. Blezinger, found alcohol, as well as salicylic acid, in quantities of 10 to 15 grams ($\frac{1}{3}$ to $\frac{1}{2}$ oz.) to cause the rapid disappearance of trichinæ. Thinking to be able to preserve human flesh and pork, containing trichinæ, by means of salicylic acid, he sprinkled the latter, in powder, over the specimens, but found shortly afterwards that the trichinæ had entirely disappeared.—*Neueste Erfind. und Erfahr.*

If this information is authentic, it might be worth while to try the effects of salicylic acid upon persons infected with trichinæ.

---

**Blistering for Neuralgia.**

℞

Chloroform,

Bay rum, of each, equal parts.

Sig.—Shake well before using.

Dr. Walker says of it in the *Southern Clinic:* "My attention was called to this as a remedy for neuralgia of the scalp some ten years ago. I have frequently used it since, and I have known it to be used by others. By applying it to the temple or seat of pain, with the end of the finger, holding the finger to the parts to exclude the air and prevent evaporation, in two or three minutes it so reddens the skin, and the pain from it is so severe, I have had to remove it to prevent its blistering. A blister can be formed, I am satisfied, in five or ten minutes. I knew a friend who applied it upon a handkerchief to his temple, while suffering with neuralgia, and becoming eased, fell asleep without removing it, though it caused a severe and troublesome blister."

---

**Diarrhœa.**

S. P. Hubbard writes to the *Medical and Surgical Reporter*, as follows :

In your journal of December 20 is an article on coto bark in the diarrhœa of phthisis. Now, I see no reason why we should resort to foreign productions when we have an abunddance of good remedies at home. Such a one you will find in the bark of the root of ceanothus, or Jersey tea. I have used it extensively for the last thirty years, and I have never had it fail me once. It is also a superior remedy in bronchial catarrh and all throat and lung affections; and in aptha or thrush I have never seen its equal. While in the army, I found it the best remedy for chronic diarrhœa, combined with opium, at night. I make a strong decoction, in an earthen vessel, sweeten it with loaf sugar or honey, and give as a common drink. A weak decoction, made in the same manner, given to infants, makes an excellent remedy in teething diarrhœa.

---

**Glaucoma.**

Dr. Thos. R. Pooley, in a brief paper, gave the results of his experience in the use of *sulphate of eserin.* especially in the treatment of acute gloucoma. He had used it in four cases. Its great value consisted in the power it had to produce temporary improvement, by which the patient could be prepared for an operaation.

Dr. H. D. Noyes, of New York, remarked in way of caution, with reference to expecting too much from the use of the remedy. In one case he had employed it without producing any benefit whatever; yet he regarded it as a valuable remedy for controlling this severe and important disease.

With reference to sympathetic glaucoma, we had not sufficient data to enable us to arrive at definite conclusions.

Dr. Knapp, of New York, referred to a case of glaucoma, in which a cure was affected by the use of eserin. The strength of the solution he used was one grain to three or four drachms of water and employed three or four times a day.—*Trans. Am. Oph. Soc.*

**Disease of the Bladder.**

Dr. Du Fau, (Le Progres Medical,) translation in *Practitioner*, recommends the *stigmas of maize* in the treatment of these ailments. In acute traumatic cystitis as well as in blennorrhagia, they exert a marked diuretic action, with an increase of pain. In such cases therefore, it is better *not* to employ the drug. The best results from its use are obtained in gravel, either uric or phosphatic, or in chronic cystitis, and in muco-purulent catarrh of the bladder. Under its use, in these cases, vesical pain, difficulty in passing water, the excretions of dark colored urine and the ammonical odour rapidly disappear. Retention gives way to the treatment, but frequent application of the catheter is recommended, that the bladder may be kept entirely empty. The syrup of the stigmas of maize is recommended. Dose two or three tablespoonfuls a day.

---

**Quinine for Children.**

It is probable that a very large proportion of the sulphate of quinine prescribed for the diseases of children is not administered as prescribed. The child objects to it on account of its bitterness, the nurse neglects to give it on account of the child's objection, the doctor does not observe the effects which he had anticipated, and is disappointed. Fortunately, the difficulty may be entirely overcome by the substitution of the neutral tannate of quinine for the sulphate. Five grains of the former equal two grains of the latter. The neutral tannate, moreover, is thought to be not inferior to the sulphate. It is tasteless, insoluable in water, and should be given in syrup or jelly. Its adoption entirely obviates all of the usual objections to the administration of quinine for children. It is a matter of suprise that its use is not more nearly universal.—*Chicago Med. Gazette.*

---

**Anti-Neuralgic.**

Allow me to mention that in neuralgia of the face, I have several times applied a solution of menthol, a solid derived from the Chinese or American oil of pepermint, referred to in *The Lancet* of 7th June last, as a powerful antiseptic and theoretically anti-neuralgic agent. The solution used was, on a first trial, one of the melted crystals only; but to avoid the annoyance to the eyes from the extreme volatility of the remedy, and to obtain a more prolonged action, I afterwards used a mixture of menthol in rectified spirit, with the addition of a little clove oil, to a strength of one of menthol to sixty parts, painted over the affected tract. Relief was had in from two to four minutes, and within one or two minutes at most after this the then existing attack was cured. This, I think, goes far to show that the Chinese custom of painting with oil of peppermint in neuralgic cases owes its reputed efficacy to menthol as its active constituent. In cases of toothache, the pain has disappeared within a few seconds after the application of a single crystal on cotton-wool. From this it is easy to go to sciatica, and I would recommend a trial of the crystal of menthol, melted, in this affection, as well as in intercostal neuralgia, brachialgia, and nerve pains in general.—*A. D. Macdonald, in The London Lancet.*

---

**Intermittent Fever.**

P. Filatow, of Ssaransk (Gouvernment: Pensa; Russia) has for three years frequently used an infusion of the sunflower (*Helianthus annuus* L.) in intermittent fever, and has, in the majority of cases, obtained as good results as could have been obtained from quinine, The infusion is prepared by cutting the stem of the sunflower (fresh or dry) into small pieces, and macerating it for three or four days with common cognac, when it acquires the color of sherry, and the distinctive taste of the drug. The dose for adults is a tablespoonful three times a day, and it may be administered before or during the paroxysm. In recent cases recovery took place readily after one to three days; in more chronic cases, the medicine had to be given for one week, and occasionally even longer. Only a few cases resisted the remedy entirely, and quinine had to be resorted to; but in several instances even the latter failed to effect a cure.—*St. Peters. Med. Wochensch.* and *Pharm. Z. f. Russl.*

---

**Diarrhœa in Tuberculous Patients.**

The following dietetic rules for the treatment of diarrhœa in tuberculous patients are given by M. Peter, (*Lyon Med.*): 1. Diarrhœa owing to catarrh of the stomach. Give subnitrate of bismuth dissolved in a little water. Strict diet. If cod-liver oil disagrees with the patient, koumiss must be given in its stead. A teaspoonful of lime-water must be added to every cup of milk which the patient drinks. 2. Diarrhœa caused by excess of food and insufficient digestion. Give one and a half grammes of ipecac, to cause vomiting, and a purgative the next day. The diet must be carefully regulated. 3. Diarrhœa owing to gastro-enteritis caused by ulcerations. Strict diet; very little milk, a soft boiled egg without bread, or beaten up in beef-tea, raw grated meat, about 20 grammes at a time, blisters on the epigastrium, bismuth, diascordium, theria, laudanum. The skin of the abdomen must be rubbed with Cologne water, alcohol, etc., or large blisters applied. Nitrate of silver may be given in pills, containing 1 centigramme each, increasing gradually to 5 centigrammes. Graves even went as far as 25 centigrammes *pro dosi.—London Med. Record.*

---

**Rosacea of the Face.**

Dr. Hillairet, in *Annales de Dermatologie*, recommends the following, which he has used with excellent success:

Wash the face several times with very warm water, and then—

℞

Sulphuris sublim, ℥ j.

Tinct. camphoræ, ʒ ij–iv.

Etherices sulphurici, ʒ j.

Aquæ, ad ℥ viij.

M. Bathe the face at night with this and let it dry on. In the morning wash, and apply—

℞

Zinci oxidi, ʒ ss–j.

Unguenti petrolei, ℥ j.

Improvement begins in a week, but the treatment should be continued several months.

---

**New Anthelmintic.**

The *Ocymum Basilicum*, a plant known in Buenos Ayres, under the name "albochaca," has an action of such a nature, that the worms in every stage of development rapidly leave their location after the juice reaches them. Its use is so much the more to be recommended since in the event of no worms being present, no injurious effect results from the plant, but merely a laxative and disinfectant action. Fifty grammes of the juice is given, followed in two hours by a dose of castor oil. A free discharge of the worms may be expected.

The above observations of Dr. Lemos and the results are very encouraging and invite further investigation, the more since the number of anthelmintics is limited, and their action often unsatisfactory.—*Med. Neuigk.*

**Whooping Cough.**

Dr. J. J. Caldwell's mode of treating this disease is to place a steam atomizer in a position on a table before the patient, charged with the following mixture:

℞

Extracti belladonæ fl., gtt. vi.–vij.

Ammonii bromidi, ℈i.

Potassii bromidi, ℈ij.

Aquæ destillatæ, fl. ℥ ij.

The spray is rapidly carried over into the face, mouth, and lungs of the child, and applied ten to fifteen minutes, until the pupils are dilated by the effects of the belladonna mixture. The applications are made morning, noon and bedtime. This has, it is said, cut short the spasmodic cough within two or three days uniformly, and almost to a certainty.—*Br. Med. Jour.*

---

**How to Gargle the Throat.**

Dr. Lowenburg recommends the following method of gargling the naso-pharyngeal cavity. The patient inclines the head horizontally backward, and performs movements which we may call "quasi-deglutition," not including the last portion of this physiological action, definite swallowing. The liquid is passed much higher behind the soft palate than the ordinary method of gargling will permit. Some persons succeed so well in this manœuvre that they are able to eject by the nose, the liquid which has been received by the mouth. Moreover, these rapid muscular contractions completely detach the abnormal secretions, which can then be easily expelled, and the greatest possible relief is thus given to the patient. Another method is to fill the mouth with the tongue; this confines the gargle to the pharynx. The head should then be bowed in a forward direction until the top of the cranium is its lowest portion. In this position the gargle will not only wash the roof of the pharynx —giving a sort of sitz-bath, as it were—but if the patient has caught the trick, will flow forward through the nose.—*Boston Medical and Surgical Journal.*

---

**Vaginismus.**

M. Gallard, in the *Annales de Gynecologie* states that he constantly recommends the gradual dilatation of the vagina by tents of progressively increasing size. According to the circumstances of the case he impregnates these tents with different applications. He also believes that these topical applications aid materially in curing vaginismus. For this purpose M. Gallard recommends the use of iodoform made up into an ointment (iodoform, 2 grams, cocoa butter, 2 grams, fresh lard, 15 grams.) This preparation may be employed when there is redness or excoriation of the mucous membrane. If there is only pain without any visible change in the mucous membrane, extract of belladonna, 2 grams, fresh lard, 15 grams, may be prescribed. In this, as in the previous case, the tents may be as small as possible. After the employment of the iodoform ointment it is well to replace it after a few days, when the redness and excoriations have disappeared, by the belladonna preparation. In both cases, care should be taken to increase daily, by an imperceptible but still advancing gradation, the size of the tent. In effecting this result, the action of the narcotic substance and the progressive dilatation have both materially assisted each other.—*From Journ. de Med. et Chir.*

---

**The Hypodermic Syringe.**

This little instrument has become almost indespensible to every practitioner of medicine. The instances must be rare where the physician has not been perplexed, not to say vexed, when called upon to administer an anodyne to a suffering patient, to find that the piston of the syringe had become perfectly dry, necessitating the loss of much precious time in restoring it to a working condition. Numerous complaints have come to us of this provoking difficulty with the instrument, and we have suffered the same inconvenience more than once. Our medical readers will thank us for the information that we have entirely overcome this exasperating abomination. We sent our syringe to George Tiemann & Co., 67 Chatham Street, New York, with directions to make us a hard-rubber case, just large enough to receive the instrument. This case we fill with a mixture of one part of glycerine to two of water, into which we plunge our syringe, (the superfluous fluid escaping from the case as the instrument is inserted,) and then screw on the top. It is now air tight, entirely preventing the evaporation of the moisture from the piston, and holding the syringe *always* ready for use. Should you not have occasion to use it for six months or a year, it will be in working order when you are called upon to remove it from its water bath. The rubber case occupies but a trifle more room than the syringe, and is carried in the usual Morocco case, with the points and bottle of morphine solution.

Geo. Tiemann & Co. supply them. [*Editor Bistoury.*]

---

**Hemorrhage at the Umbilicus in Infants**

Occurs once in every three thousand to four thousand cases. If it be arterial or venous, which is exceedingly rare, the bleeding vessels may be ligated by the ordinary method; but generally the hemorrhage is parenchymatous, and the oozing is so profuse that, unless arrested, fatal syncope ensues in twenty-four or thirty-six hours. The usual treatment by bandaging, by pressure, or by the application of styptics, generally fails. A suture entered on one side of the umbilicus, passed underneath the oozing surfaces, brought out at a corresponding point on the other side and tightened, rarely arrested the hemorrhages permanently, since it generally reappears along the line of the suture. A certain, safe and efficient remedy is, however, always at hand. It may be summed up in the three words, ligature en masse. The ligature should be applied as follows: Pass a long, slender needle far underneath the bleeding part, entering about one-third of an inch from the margin of the umbilicus on one side and emerging at the corresponding point on the other. Pass another needle in the same manner at right angles to the first. Secure a strong silk ligature underneath the two needles. This will arrest the hemorrhage for a time in all cases, permanently in most. Should hemorrhage recur, the needles may be withdrawn, and again introduced in other directions, and the ligature applied as before. This may be repeated until the hemorrhage has permanently subsided.—*Chicago Med. Journal.*

---

**Supra-orbital Neuralgia Cured by Nerve-stretching.**

Dr. Kocher relates, in the *Correspodenzblatt fur Schweizer Aerzte*, November 11th, 1879, the case of a man aged 32, who had for seventeen years suffered from neuralgia of the right supra-orbital nerve. The attacks, at first rare, afterwards became more frequent, until at last there were only brief intervals of freedom from pain. All the ordinary therapeutic measures had been tried for years without success. Dr. Kocher laid bare the nerve and three of its branches by an incision along the upper border of the orbit, and stretched it forcibly by means of an aneurism-needle passed under it. The healing of the wound was attended with abundant suppuration. From the moment of the operation, the patient was free from pain, and the neighborhood of the supra-orbital nerve was anæsthetic. The patient was last seen three months after the operation; he had had no return of the pain; sensation was diminished over a space ten *centimetres* in extent, but was otherwise perfectly restored. After neurectomy, paroxysms of pain are usually observed during the first few days after the operation. As these were absent in the present case, Dr. Kocher concludes that the lesion of the nerve is less when the nerve is stretched than when it is divided. The value of nerve-stretching as a substitute for excision will be greater in neuralgia of the second and third divisions of the fifth nerve, as here a much smaller wound will suffice.– *British Med. Journal.*

---

**Goitre.**

Dr. Wolfler, in speaking of the treatment of goitre with subcutaneous injections of iodine, says (Langenbeck's *Archiv*, Bd. 24, Heft. 1) that favorable results have been obtained both in case of simple hyperplasia, and of colloid degeneration. He illustrates his statement by a few cases from Billroth's clinic, and an experiment on a dog made by himself. The lobes of the thyroid gland of this dog had respectively attained the size of a goose's egg, and the author made ten injections of iodine into one of the lobes. The dog was killed at the end of a month, when the portion of the goitre into which the injections had been made was found to have dwindled down to the size of a man's thumb; it consisted of connective tissues which no longer contained any colloid liquid. The peripheric part of the injected goitre presented the same appearance as the lobe which had remained untouched; it consisted of large meshes of connective tissue, which contained colloid fluid. There were no traces of inflammation or hemorrhage following the injection of iodine. Several strumous cysts were treated in a different manner; one cyst with thin walls was absorbed after injections of iodine; two other cysts resisted this treatment. In two cases Billroth drained strumous cysts with antiseptic precautions. In one of these cases, the cure was speedily effected; in the other, the cyst was not wholly absorbed, as there were calcareous deposits in its walls. The sac was then opened and the contents removed, after which the patient, a woman aged 72, recovered. The author thinks that tapping the cyst and putting in a drainage tube ought to be done in cases where a cyst does not collapse immediately after being tapped, or in old people where the injection of iodine might be succeeded by a too strong reaction, but where extirpation of the goitre might prove fatal. In the course of the last year, Billroth has extirpated goitres in seven cases under antiseptic precautions, the results having each time been very favorable. In one of these cases the patient was suffering from malignant cystous papilloma; in another case the struma was of carcinomatous nature. All the wounds healed by first intention.—*London Med. Record.*

---

**Milk Diet for Articular Rheumatism.**

M. Bigot, in the *Revue Mensuelle de Medecine et de Chirurgie*, gives a summary of the clinical facts scientifically observed at the Hotel Dieu, at Lyons, on this subject. The deductions and conclusions drawn by M. Biot touching the nature of acute articular rheumatism, and the efficacy of the milk regimen in the course of this affection, are based on a number of analyses of urine made as completely as possible, since they give the amount of the total nitrogen, of the urates, of the total chlorides, and of the phosphuric and sulphuric acids. His theoretic and therapeutical views on the subject are thus summarized :

The fever of acute rheumatism generally lasts two or three weeks, and consequently, either from the time it lasts or on account of the rise of temperature, causes an enormous consumption of blood corpuscles, which produces profound anæmia in the patient. The fall of temperature is the best criterion of the cure, and coincides exactly and constantly with the disappearance of the pains. The tortures endured by patients suffering from articular rheumatism are in themselves alone of a violence and tenacity sufficient to induce the physician to endeavor to oppose to this disease a treatment which would unite the three qualities, *Cito, tuto, et jucunde.* The milk diet seems capable of fulfilling this desideratum; it causes the temperature to fall rapidly below hyperpyrexia, and simultaneously assuages the pains in a period varying from three to eight days. The effects from these two points of view are more prompt and more powerful if the patient be submitted to the milk regimen at the outset of the affection. This milk regimen, without overcharging the stomach or raising the temperature, by its nutritive power and its facility of digestion, prevents in great measure that characteristic and generally troublesome anæmia left behind by attacks of rheumatism. Besides these general effects milk diet has a special action on the urinary function, which is clearly indicated in rheumatism. Milk strongly favors the elimination of all the waste principles accumulated in the organism; its exclusive use causes both the quantity of urine excreted in twenty-four hours and the quantity of all the saline principles dissolved in this liquid, to increase rapidly; density, on the contrary, experiences a proportionate decrease. The impetus given to the urinary function by a milk regimen allows a glimpse of the nature of rheumatism, its near and intimate causes. The analysis of urine seems to show that there is an accumulation of urates or uric acid in the organism of rheumatic sufferers, and that its diminution under the influence of milk is not one of the smallest benefits of this regimen.—*British Med. Journal.*

---

**Bloodless Method of Operating**

With the elastic bandage of Esmarch, has, in some cases, a disadvantage. The smaller vessels may remain unligated, and after the removal of the elastic tube, the suddenly augmented pressure and consequent dilatation may produce bleeding sufficient to destroy any advantage gained by the small loss of blood during the operation. Its use has also been charged with increased liability to secondary hemorrhage. Prof. Konig, of Gottingen, claims to be able to prevent both of the foregoing hemorrhages. His plan is as follows: After the cutting has been completed, all the larger arteries that can be found are to be ligated; the part operated upon is then to be held up in a vertical position, then the compressing elastic tube is to be removed. If, for instance, the operation has been on the foot, an assistant is to elevate the leg to the vertical position before the removal of the elastic tube. The subsequent hemorrhage is then very slight. The vertical position is to be maintained during the closing and bandaging of the wound, and also during the removal of the patient to the bed. During the following two or three days a nearly vertical position is to be maintained, either by a suspensory apparatus or an inclined plane—the back of an inverted chair, along which the extremity may be secured on a pillow, is a serviceable device.—*Chicago Med. Gazette.*

---

**Urticaria.**

Dr. L. Duncan Bulkley, in *Archiv. Dermatology*, says that in the treatment of urticaria, he has commonly afforded much relief by the external application of a tolerably weak solution of bicarbonate of soda (ʒ ij. to vj. to the pint) with a little glycerine, the surface to be bathed with this morning and night, and subsequently lightly dusted with a starch or rice powder. Carbolic acid (ʒ j–ʒ iv. to the pint) gives much relief. The *liquor picis alkalinus*, diluted with ten to twenty parts of water and used as a wash, will often afford perfect relief. The formula for this preparation of tar is:

Tar, 2 drachms.
Caustic potassa, 1 drachm.
Water, 5 drachms.

Dissolve the potash in water and add slowly to the tar in a mortar with friction. Baths are often of the greatest service, especially the *alkali and starch bath*. This is made as follows:

Carbonate of potassa, 3 ounces.
Carbonate of soda, 2 drachms.
Powdered borax, 1 ounce.

Mix. Use one such powder for a thirty-gallon bath, with from one-quarter to one-half pound of starch. The surface may afterwards be anointed with cosmoline, containing from five to ten grains of carbolic acid to each ounce. When the itching is uncontrollable, the *chloral camphor ointment* will surely give relief. This is prepared thus:

Chloral hydrate, 1 drachm.
Camphor, 1 drachm.
Rose ointment, 1 ounce.

Rub well together the camphor and chloral in a mortar until a liquid results, and add to it the rose ointment. It should not be forgotten that irritating underclothing may excite and keep up urticaria, and in severe cases silk garments should be worn next the skin, or a very thin muslin may be interposed beneath a woolen shirt or drawers. In addition to the local treatment, hygienic and dietetic as well as constitutional treatment should be employed.

